# Advanced Cell Therapies for Glioblastoma

**DOI:** 10.3389/fimmu.2022.904133

**Published:** 2022-08-16

**Authors:** Guangwen Wang, Wenshi Wang

**Affiliations:** ^1^BlueRock Therapeutics, Department of Process Development, Cambridge, MA, United States; ^2^Metagenomi Inc., Department of Cell Therapy, Emeryville, CA, United States

**Keywords:** glioblastoma, cell therapy, immunotherapy, clinical trials, CAR T cells, NK cells, myeloid immune cells, stem cells

## Abstract

The sheer ubiquity of Gioblastoma (GBM) cases would lead you to believe that there should have been many opportunities for the discovery of treatments to successfully render it into remission. Unfortunately, its persistent commonality is due in large part to the fact that it is the most treatment-resistant tumors in adults. That completely changes the treatment plan of attack. Long established and accepted treatment therapies such as surgical resection, radiation, and aggressive chemotherapy, (and any combination thereof) have only confirmed that the disease lives up to its treatment-resistant reputation. To add to the seemingly insurmountable task of finding a cure, GBM has also proven to be a very stubborn and formidable opponent to newer immunotherapies. Across the board, regardless of the therapy combination, the five-year survival rate of GBM patients is still very poor at a heartbreaking 5.6%. Obviously, the present situation cannot be tolerated or deemed acceptable. The grave situation calls for researchers to be more innovative and find more efficient strategies to discover new and successful strategies to treat GBM. Inspired by researchers worldwide attempting to control GBM, we provide in this review a comprehensive overview of the many diverse cell therapies currently being used to treat GBM. An overview of the treatments include: CAR T cells, CAR NK cells, gamma-delta T cells, NKT cells, dendritic cells, macrophages, as well stem cell-based strategies. To give you the complete picture, we will discuss the efficacy, safety, and developmental stages, the mechanisms of action and the challenges of each of these therapies and detail their potential to be the next-generation immunotherapeutic to eliminate this dreadful disease.

## Introduction

To illustrate the daunting task a researcher is up against with Glioblastoma, one just needs to state a few facts about GBM. In most medical cases, just one fact would give you pause - but with GBM it is a perfect storm of resistance. Consider this: GBM is the most common primary brain tumor in adults. In fact, it comprises over half (51%) of all gliomas (1). And to continue the bleak outlook, it is also the deadliest primary brain tumor in adults. Every year, it accounts for about 10,000 deaths in the US alone. It is also the most aggressive. Even with the current standard of care, combined therapies including resection, radiation therapy (RT), and chemotherapy with temozolomide (TMZ), median overall patient survival rate is a mere 14.6 months from diagnosis (2). Which means for the patient, there is little hope of a prolonged life or even quality of life, let alone a full recovery. Just to treat the existing GBM tumor, the treatment process brings with it adverse and often morbid effects of RT and chemotherapy. And if luck somehow prevails and the tumor is removed or contained, the disease almost always recurs and is inevitably fatal. The five-year survival rate remains a dismal 5.6% (3). The challenge of GBM is its near complete resistance to current standard treatment options. Regardless of the treatment strategy pursued, difficulties arise. If surgery is the option, brain surgery of course is a gauntlet of potential pitfalls in any attempt to fully resect the tumor. GBM also displays resistance to radiation and chemotherapy, resulting in GBM recurrence (4). If any progress is made, it is negated by the rapid growth rate of the returning tumors (5). The existence of the blood–brain barrier (BBB) also adds a layer of difficulty because BBB reduces the bioavailability of systemically administered drugs within the central nervous system (CNS) (6). To compound the treatment difficulties, the established therapies mentioned above are joined by the newer immunotherapies in their ineffectiveness against GBM resistance. Some immunotherapies which have improved outcomes in other types of cancers are incapable of impacting on GBM’s clinical outcome. This is due to GBM being among the immunologically “coldest” tumors, characterized by high intratumoral and intertumoral heterogeneity, low mutational burden, highly invasive and infiltrative GBM cell properties, systemic immunosuppression and the local severely immunosuppressive tumor microenvironment (TME) promoting GBM growth (7–9). Another critical factor that needs to be taken into consideration in GBM treatment is that Glioblastoma Stem Cells (GSCs) are resistant to all standard therapies with potent tumor regenerative power (5, 10). To state the obvious, there is a desperate need to develop some innovative and more effective therapeutic strategies to improve the outcome of GBM treatments and increase the life expectancy of GBM patients.

Attempting to answer the urgent call is work being done in the adoptive cellular therapy (ACT) area. ACT is a rapidly growing area of immunotherapy and clinical investigation. Various immune cells and stem cells have been investigated, developed and applied to treat GBM. In this review, we provide a comprehensive overview of some diverse cell therapies in treating GBM, including CAR T cells, CAR natural killer (NK) cells, gamma-delta T cells (γδ T) cells, natural killer T (NKT) cells, dendritic cells (DC), macrophages with clinical experience summarized in **Table 1**, as well as stem cell-based strategies with available trial information summarized in **Table 2**. We summarize the efficacy, safety, and development stages of these cell therapies, discuss their mechanisms of actions, the hurdles that these therapies face with possible improvements as well as the potential and future directions of these nascent cell therapeutic modalities as the next-gen immunotherapies for GBM treatment.

**Table 1:** Clinical trials of immune cell-based glioblastoma therapy with clinical outcomes.

Clinical trial ID	Cell type	Modifying factor	Trial phase	Route of administration	Clinical Outcomes
NCT00730613	T cell (Autologous)	CAR-IL13Rα2	Pilot	ICT, ICV**	Pilot trial: No dose-limiting toxicities were observed. Transient clinical activity was observed in two of 3 patients.
NCT02208362	T cell (Autologous)	CAR-IL13Rα2	I	ICT, ICV**	Phase I: Regression of intracranial and spinal tumors was observed, 7.5-month regression period with a median overall survival of 11 months
NCT02209376	T cell (Autologous)	CAR-EGFRvIII	I	intravascular	No evidence of off-tumor toxicity or cytokine release syndrome. One patient of the 10 patients showed stable disease for over 18 months of follow-up.
NCT01454596	T cell (Autologous)	CAR-EGFRvIII	Pilot	intravascular	One patient developed fatal and another patient serious respiratory symptoms shortly after cell infusion at the highest dose levels. One patient was still free from disease progression 6 months after therapy
NCT01109095	T cell (Autologous)	CAR-HER2 T	I	intravascular	The treatment was well-tolerated, without dose-limiting toxicities. Limited clinical benefit was observed. One patient out of the 16 had a partial response lasting 9 months, seven had stable disease, and eight progressed after CAR-T cell infusion. In the 24 to 29 months follow-up, three patients with a stable disease status were alive without any evidence of progression of the disease.
NCT01082926	T cell (Allogeneic)	GRm13Z40-2: CAR - IL13Rα2 with GR disrupted	I	intracranial	No graft-versus-host alloreactivity. Transient anti-tumor effects in four of the six treated GBM patients
*KCT0003815	NK cell (Autologous)	no	I	intravascular	Safety demonstrated with grade 1or 2 common side events. Median OS was 22.5 months, and the median progression-free survival was 10 months
NCT04165941	γδ T (Autologous)	MGMT	I	intravascular	Safety: No dose limited toxicity was observed. First patient survived 15.6-month post diagnosis.
NCT00846456	DC (Autologous)	GSC-mRNA	I/II	intradermal	No adverse autoimmune events. Progression-free survival was 2.9 times longer in vaccinated patients comparing with controls.
NCT01280552	DC (Autologous)	ICT -107: Synthetic peptides (MAGE-1, HER-2, AIM-2, TRP-2, gp100, and IL13Rα2) pulsed DC	II	intradermal	Median overall survival favors ICT-107 by 2.0 months; significant increase of PFS (2.2 months)
NCT01006044	DC (Autologous)	DCVax^®^-L:DC pulsed with tumor lysate	II	intradermal	Median PFS is 12.7 month; Median OS is 23.4 months
NCT03395587	DC (Autologous)	DCVax^®^-L	III	intradermal	mOS is 23.1 months
NCT00639639	DC (Autologous)	CMV pp65-LAMP mRNA	I	intradermal	Median PFS was 25.3 month, and the median OS was 41.1 months. 4 patients were free of progression after 59 to 64 months from diagnosis

*Case is from the Korea Clinical Research Information Service database.

**ICT, intracranial at the tumor site; ICV, intracranial into the ventricles.

**Table 2:** Clinical trials of stem cell-based therapy to treat glioblastoma.

Clinical trial ID	Cell type	Cell product name (modifying factor)	Trial phase	Route of administration	Status
NCT04657315	MSC (not specified)	MSC11FCD (Cytosine Deaminase)	I/II	Intratumoral	Enrolling by invitation
NCT03896568	MSC (allogenic)	Ad5-Delta24RGD (Oncolytic Adenovirus Ad5-DNX-2401)	I	Intra-Arterial	Recruiting
NCT02055196	NSC (allogenic)	hCE1m6-NSC (carboxylesterase)	I	Intracerebral	Withdrawn (New study written)
NCT02015819	NSC (not specified)	HB1.F3.CD NSC (Cytosine Deaminase)	I	Intracranial	Completed
NCT03072134	NSC (not specified)	NSC-CRAd-Survivin-pk7 (oncolytic adenovirus)	I	not specified	Completed
NCT01172964	NSC (not specified)	HB1.F3.CD NSC (Cytosine Deaminase)	I	Intracranial	Completed
NCT02192359	NSC (allogenic)	hCE1m6-NSC (carboxylesterase)	I	Intracranial	Recruiting
NCT05139056	NSC (not specified)	NSC-CRAd-S-pk7 (CRAd-S-pk7)	I	Intracerebral	Not yet recruiting
NCT05052957	Hematopoietic Progenitors (autologous)	P140K-MGMT	II	infusion	Not yet recruiting

## Chimeric Antigen Receptor (Car) T Cells

The most mature and developed gene modified cell therapy for cancer is CAR T cells therapy (11). CAR T cells therapy refers to ACT of human T cells genetically modified to stably express the CAR. The CAR is composed of an antigen recognition domain of a specific antibody and intracellular T cell activation domain. The CAR expressed on T cells allows T cells to activate and function bypassing the MHC restricted TCR signaling (12, 13). The ACT of CAR T cells has claimed promising clinical activity in a subset of cancers, particularly in B cell malignancies (14, 15). CAR T therapy has offered the cure for two patients with chronic lymphocytic leukemia. These two CAR T treated patients achieved complete remission in 2010 and have sustained this status until now (16). CAR T cells therapy has been approved for the treatment of lymphoma and leukemia in multiple countries (17).

This success has inspired similar methods to target GBM, engineering patients’ T cells with CAR constructs to recognize selected tumor antigens/tumor related antigens that are overexpressed in GBM and have little to no expression in healthy brain tissue or elsewhere in the body. This targeting method reduces the risk of healthy brain tissue suffering compromising effects from the therapy. Selected antigens include interleukin-13 receptor alpha 2 (IL13Rα2) (18, 19), epidermal growth factor receptor variant III (EGFRvIII) (20), human epidermal growth factor receptor 2 (HER2) (21) and erythropoietin-producing hepatocellular carcinoma A2 (EphA2) (22). Researchers are also looking at newly developed targets to study including ganglioside 2 (GD2) (23), B7-H3 (24, 25) and chlorotoxin (26). All of these have demonstrated promising preclinical results (27). Some of these targets have been evaluated in clinical phase I, phase I/II and phase II trials. Several newer targets, such as CD70 (28, 29), CD133 (30) and MET (the receptor of hepatocyte growth factor (HGF) (31, 32), are in the preclinical investigation stage. Cytolytic activity has been demonstrated against GBM cells (33).

Human T cells are lymphocytes that belong to the adaptive immune system. So far, majority of the GBM CAR T therapies have been autologous treatments with single or multiple dose (3-6) infusions. This approach features the delivery routes of CAR T cells which are intracranial at the tumor site (ICT), intracranial into the ventricles (ICV) and intravascular (IV) administration (34). These cell therapies have shown early promise in multiple examples of clinical feasibility for GBM (**Table 1**). There is evidence of safety given the fact that there are no signs or symptoms of off-tumor toxicity or cytokine release syndrome for the treatment of recurrent GBM. This compromising effect was observed in some patients with preliminary clinical activity and transient responses (34, 35). In 2016, Dr. Brown et al. reported a GBM patient achieved a complete clinical response and significant regression with IL13Rα2-specific CAR T treatment. An MRI did not detect either intracranial or spinal tumors. For approximately 7.5 months, after the initiation of treatment, both the intracranial and spinal tumors experienced regression. The researchers did not observe dose-limiting toxicities (19). In the following year, Dr. O’Rourke from the University of Pennsylvania published their report from a Phase I clinical trial. In this trial, researchers evaluated EGFRvIII-specific CAR-T therapy in 10 patients suffering from recurrent glioblastoma. The patients received a single dose of up to 5 × 10^8^ autologous EGFRvIII-specific CAR-T cells administered as an IV injection. No sign of off-tumor toxicity or cytokine release syndrome was not observed or reported. A single patient showed no disease progression for over 18 months (20). At the National Cancer Institute, the third generation of EGFRvIII-specific CAR-T cells were dosed in 18 patients with recurrent EGFRvIII-positive GBM. Not a single objective response was observed. There was one patient free from disease progression status 6 months after the therapy. However, at the highest dose level, one patient died and another patient developed serious respiratory symptoms shortly after the infusion (36). In a trial published in 2017 by Ahmed et al, some very interesting results were reported. In this Phase I dose-escalation trial, 17 patients who were diagnosed with progressive ErbB2-positive GBM received an IV infusion of one or more doses of up to 10^8^/m^2^ autologous ErbB2-specific CAR-T cells. The patients’ acceptance level of the infusions was “well-tolerated” due to the absence of any toxicities that might limit the strength and number of dosages. The results were reported for 16 of the 17 patients that were involved. Of the 16 patients, one had a partial response that lasted nine months, and seven patients showed stable disease. In the eight remaining patients the disease had progressed after the CAR-T cell infusions. In the 24 to 29 months follow-up time period, three patients with a stable disease status were alive without any evidence of progression of the disease (21). Earlier this year, Stanford University published the clinical outcome of a GD2-CAR T cells phase I dose-escalation trial. Children and young adults afflicted with pontine and spinal cord diffuse midline gliomas were given CAT that contained a GD2 binding domain, a 4-1BB co-stimulatory and a CD3ζ domain to target the H3K27M mutation. The H3K27 mutation refers to a K27M mutation in genes encoding histone H3. Three out of the first four patients revealed clinical and radiographic improvement without on-target, off-tumor toxicity (24). This trial also provides a solid rationale for applying GD2-CAR cell therapy to treat H3K27M-mutated GBM.

CAR T cell therapy has shown some promising preclinical efficacy and limited clinical responses in GBM, as demonstrated by a low level of anti-tumor response. However, there are significant challenges in using this therapy for treating GBM. Obstacles include the tumor heterogeneity; antigen loss, escape and downregulation (37); and hostile immunosuppressive tumor microenvironment. Current CAR T therapy for treating GBM uses autologous T cells. Since GBM patients’ T cells might have already acquired impaired immune signals/characteristics, these signals/characteristics might contribute to the modest clinical outcome seen with this therapy (38), suggesting that allogeneic approaches may be more successful as cell therapy approaches advance.

To induce more efficient anti-tumor immune responses, new strategies are being developed. Specifically, the new strategies include the identification of novel tumor-specific targets, and the engineering of CAR T cells to achieve multi-specificity. Two of these are engineering the bi-and tri- specific lower antigen responding CARs. Bi-CARs or Tri-CARs can target multiple GBM surface antigens simultaneously. The objective is to induce more efficient anti-tumor immune responses and prevent tumor antigen escape (39, 40), and the preclinical results have been promising. The new TanCAR (Tandem CAR) joins a HER2-binding scFv and an IL13Rα2-binding IL-13 mutein (41) or TanCAR cognizes IL13 (4MS) and EphA2 scFv (42) have been tested in the pre-clinical xenograft mouse model. The TanCAR demonstrated a more efficient and selective killing than single CAR. The TanCAR can potentially decrease off-target cytotoxicity and reduce the possibility of antigen escape.

To overcome the immunosuppressive microenvironment of GBM, a variety of approaches are being investigated. It has become evident that physical barriers and stromal and immune cells in the tumor microenvironment, which express and release an array of immunosuppressive molecules, limit CAR-T cell persistence and efficacy. In these hostile immunologic circumstances, researchers are employing promising strategies aimed at remodeling the tumor microenvironment or conferring intrinsic CAR-T cell resistance to immunosuppression (43). In recent studies, most CARs include costimulatory signaling domains to increase the T-cell activation, survival and/or function. These have been named “armored” CAR-T cells, which express proinflammatory cytokines (IL-12) or a combination of CAR-T cells with oncolytic viruses (44). Additionally, gene ablation is a technology that has been shown to allow CAR-T cells to avoid immunosuppressive signals in the TME. The combination of biologics, such as checkpoint inhibitors are expected to improve the effectiveness of this new GBM treatment. Combining immunotherapeutic reagents, such as PD-1 checkpoint inhibitors to enhance adoptive CAR T-cell therapy are now being broadly investigated (34).

Safety is still at the forefront of concerns for GBM treatment. The sensitivity of the CNS to inflammation and an immune response is paramount. To date, CAR T-cell trials in GBM have not shown severe CRS and neurotoxicity-like adverse events. While we further optimize the potency of CAR T therapy, we will gain understanding of the full toxicity profile of GBM CAR T-cell therapy (34). Promising, regional administration (ICT and/or ICV) of CAR T cells effectively restricts peripheral tissue toxicities.

One drawback of autologous CAR T cells therapy is that it can not be used on patients immediately upon diagnosis. This is because the autologous therapy requires bespoke manufacturing for each and every patient which also lead to the high cost of this therapy. A very recent clinical trial demonstrated feasibility of off-the-shelf CAR T products in GBM treatment. Allogeneic IL13Rα2-targeted CAR+ (IL13-zetakine+) T cells with a permanently disrupted glucocorticoid receptor (GR) (GRm13Z40-2) were generated from healthy donors. The resistance to glucocorticoid treatment was engineered using gene editing zinc finger nucleases (ZFNs), allowing the CAR T products to be combined with dexamethasone treatment. Dexamethasone is widely used in clinical trials to attenuate tumor-related neuro-edema and the rejection of the therapeutic allogeneic cells. In a phase I trial, allogeneic GRm13Z40-2 T cells were demonstrated to be safe and they produced a transient clinical response: four of the six treated patients experienced tumor reduction and/or tumor necrosis at the site of the T cell infusion. (45). This first-in-human trial showed the feasibility of off-the-shelf CAR T products to treat GBM which will significantly shorten the waiting time of GBM patients to receive this cell therapy treatment.

## Natural Killer (Nk) Cells & Car-Nk Cells

NK cells possess a unique biological attribute that makes a potential treatment strategy for GBM. Unlike T cells, NK cells are part of the innate immune system and can recognize and directly eliminate cancer cells. NK cell therapies have been shown to mediate the regression of solid cancer including in GBM patients (46). Herein, we will discuss the development, challenges, and potential of autologous and allogeneic NK cell and CAR-NK cell treatments.

This ability to recognize and eliminate cancer cells is done through a major histocompatibility complex (MHC)-independent mechanism that does not need to have any prior antigen experience (47). Once they are activated, NK cells can release interferon gamma (IFN-γ), perforin and granzymes, and upregulate death ligands- such as FAS ligand and tumor necrosis factor–related apoptosis-inducing ligand (TRAIL). NK cells can also initiate the apoptosis of tumor cells through a caspase pathway. They can also kill cancer cells through antibody-dependent cellular cytotoxicity (ADCC) mediated by FcγRIIIA/CD16a. NK cells regulate and activate the adaptive immune response and crosstalk with dendritic cells. This means NK cells regulate DC maturation which enhances the presentation of tumor antigen to modulate T-cell mediated anti-tumor adaptive immune responses (46). Conversely, DCs have been found to enhance the direct anti-tumor activity of NK cells. Many clinical studies support the concept that mature GBM cells can be efficiently targeted by NK cells (48, 49) and that the GBM-associated stem cells are susceptible to a NK cell–mediated killing (50, 51).

The earliest trials of GBM patients treated with NK cells have been autologous. Patients with recurrent GBM have shown durable response to ACT of ex-vivo-expanded activated autologous NK cells and T lymphocytes derived from the patients’ peripheral blood mononuclear cells (PBMC). In South Korea, an investigator recruited 14 patients between 2013 and 2017 to take part in an investigator-initiated trial. IV injections of activated NK cells were administered at two-week intervals 24 times (12 months duration). This was done after surgical resection or biopsy. The autologous adoptive NK cell therapy was shown to be safe with no adverse events at the grade 4 or 5 level, such as leukocytopenia, thrombocytopenia, brain swelling or hydrocephalus, were observed. The levels of severity of the most common adverse events considered to be treatment-related were at grade 1 or 2. Grade 1 adverse events were shown as injection site reactions, chills, hot flushes; grade 2 adverse events were amenia, anorexia, and autoimmune disorder. The most common high-grade adverse event was a fever at the grade 3 level. Median overall survival (OS) was 22.5 months, and the median progression-free survival rate was 10 months. The last follow-up to the trial occurred two years after the completion of the therapy. It was reported that five patients out of the 14 were still alive and showed no clinical decline, but they did show durable responses with enhanced immune reaction transcriptomic signatures (52). In another phase I trial, nine patients with recurrent malignant glioma received treatment; three of which had GBM. The NK cell-rich effector cells were expanded ex vivo from autologous PBMCs, and were then used to treat patients with systemic low-dose interferon (IFN). It was apparent that the NK cell therapy was safe and partially effective. Two patients experienced a partial response, two patients experienced a mixed response, and three patients experienced stable disease status during treatment (53).

Although generally safe, autologous NK cells provide limited cytotoxicity against GBM tumors. Inspired by this limited success, allogeneic NK cells are gaining more attention for therapeutic purposes. This is because they are highly cytotoxic to various cancers and display minimal risk of graft-verse-host diseases (GvHD) (54, 55). In theory, cells from screened healthy donors should be more potent and capable of eradicating tumor cells than autologous NK cells from GBM patients. This is because the NK cells from GBM patients may already express immune impairment signatures. It is important to note that GSCs appear highly susceptible to the killing ability of allogeneic NK cells (56).

Allogeneic NK cells can be sourced from peripheral blood NK cells (PBNK) from healthy donors, induced pluripotent stem cells (iPSCs), embryonic stem cells (ESC), and umbilical cord blood (UCB). Current studies have demonstrated the safety and efficacy of allogeneic NK cell ACTs as a means of treating hematologic malignancies. Some success, to a lesser extent, has been demonstrated in clinical studies with solid tumors (57–59). Allogeneic NK cell-based therapy is safe without significant GvHD. It has the potential to generate off-the-shelf cellular therapy products. Allogeneic transplantation is generally preferable, due to the bypass of the inhibitory “self”-signal displayed by MHC. Indeed, the use of allogeneic cells can greatly simplify manufacturing. Many of the obstructing issues encountered with autologous cells including the variability from patient-to-patient and the actual production time can be deciphered (60, 61).

At present, CAR-NK cells have entered clinical development for the treatment of GBM, following similar approaches taken with CAR-T cells for other tumor types. The expression of CARs can dramatically increase the NK cells’ native recognition and elimination of cancer cells. CAR-NK therapy shows promise for the development of precise and specific cancer immunotherapies. When NK cells are genetically engineered to express a CAR, they add CAR-meditated killing activity in addition to their natural cytotoxicity (58). Several groups have reported improved NK killing activity by switching the conventional T-cell CARs (CD3ζ and CD28 and/or CD137) domains with one or more NK signaling domains derived from CD244 (2B4), NKG2D, DAP10 or DAP12. This results in better NK activation and an enhanced tumor-killing function. The CAR targets that are engineered onto NK cells are the same or very similar to what we described in the previous CAR T paragraph above, where they are overexpressed in GBM cells and have little to no expression in surrounding healthy tissue. The demonstrated targets for CAR NK therapy are EGFR (62), EGFRvIII (63), HER2 (64), CD133 (65), GD2 (66) and IL-13Rα2 (67). Most of the CAR-NK therapies for GBM have moved into the preclinical and clinical development stages. In the preclinical stage, CAR NK therapies have demonstrated efficiency in orthotopic mouse xenograft models (46). In July 2020, the FDA cleared an allogeneic NK trial, but the trial was terminated in January 2022 because of a business decision (ClinicalTrials.gov Identifier: NCT04489420). This clinical trial, CYNK-001, was intended to investigate the maximum safe dose (MSD) or the maximum tolerated dose (MTD) of *in vitro* culture-expanded human placental CD34+ cells derived NK cells.

NK cell therapy for GBM faces several formidable barriers. The first is the restrained infiltration of NK cells into GBM tumor cells and GBM tumor sites. The second is the escape and downregulation of target antigens on the tumor cells, and the inhibitory cytokines, chemokines and secreted factors in the TME. It has been found that GBM develops protective mechanisms to evade NK cell-mediated oncolysis. These include disruption of receptor-ligand interactions between NK and tumor cells and the release of immunosuppressive cytokines into the microenvironment particularly transforming growth factor β (TGF-β) (68). TGF-β can be produced by glioblastoma cells, glioblastoma-associated myeloid cells, or Tregs, and represses NK cell cytotoxicity by downregulating the NKG2D activating receptor (69). These immunosuppressive cells promote additional repressive signals to diminish NK cell cytotoxicity, such as HLA-G expression (70). HLA-G is known to inhibit NK cells *in vitro* and has broad immunosuppressive activities *in vivo* (71). Some combination treatment strategies have been tested and shown promise in overcoming these obstacles in GBM. One example is the augmenting of NK cells through the increased expression of activating receptors including the natural killer cell group 2D (NKG2D). This engineered NKG2D expression can increase the anti-cancer cytolytic effects of NK cells on solid cancers (72).

The use of the synthesized cationic supramolecular inhibitor of Hsp90 (“SCI-101”) is a recent approach that could be leveraged for combined GBM treatments. SCI-101 was developed for optimal crossing of the BBB and sustaining the NK cell–activating target antigens expression on tumor cells. In the lab, the drug-resistant GBM cells were nearly eliminated by NK cells after exposure to SCI-101. This was likely due to the sustained express, over 72 hours, and the boosted ligand expression. These data suggest further investigation is needed in *in vivo* studies looking at combining NK therapy and SCI-101 paradigm for patients with GBM (46).

Another promising combined approaches is the injection of TGF-β inhibitors in combination with NK cells, which has been shown to rescue the cytotoxic capacity of NK cells and the expression of NK activation receptors NKG2D and CD16 (73). In a xenograft (PDX) orthotopic GBM mouse model derived from patient cells, the direct blockade of αv integrin or TGF-β or TGF-β receptor 2 (TGFBR2) on the allogeneic NK cells can abrogate the GSC-induced NK cell dysfunction completely, resulting in the effective control of the tumor. These data strongly recommend the regulation of the αv integrin/TGF-β axis for the NK cell therapy of GBM (74). The checkpoint inhibitors, such as anti PD-1, anti CTLA-4, anti LAG3 and anti TIGIT applied with CAR cell therapy may improve the outcomes in solid tumors (75). In preclinical models, the combination strategies have depicted the possibility of reversing the immunosuppressive impact of GBM. These combination therapies are NK cells co-delivered with an antibody that recognizes a GBM antigen or the NK cells with a histone deacetylase inhibitor (HDACi). HDACi can upregulate the expression of the NKG2D ligand (76). Interestingly, the proteasome inhibitor bortezomib pre-treatment can enable the NKG2D- or TRAIL-mediated NK-cell killing of GBM cells and improve the survival rate in animal models (77).

In summary, NK cell therapy, particularly allogeneic CAR-NK cell therapy has great promise in the treatment of GBM. Some of the promising products have progressed into clinical development. Ongoing hurdles in making these treatments more accessible are the technical challenges in NK therapy development and GMP manufacturing. Two of these challenges are the gene modification and the industrial scale expansion of functional NK cells. To develop a robust process to generate a NK product on a large scale in the GMP environment, the selection of more appropriate and effective transfection approaches and efficient expansion methods are the critical steps needed to ensure the success of NK clinical trials of GBM.

## Gamma Delta T (Γδt) Cells & Engineered Γδt Cells

γδ T cells are rare immune lymphocytes that bridge between the innate immune system and the adaptive immune system. Unlike conventional T cells expressing α and β T-cell receptors (TCRs), γδ T cells express TCRs with distinct γ and δ chains on their surface. They also express NK receptors, such as NKG2D, NKp30, NKp44 (78). The γδ T cells in peripheral blood is initially small, ranging from less than 2% of cord blood (CB) T cells to around 5% of PB T cells in adults (79, 80). However, γδ T cells are abundant in the skin, the intestines, and the liver. The majority of γδ T cells in adult peripheral blood are γ9δ2 T, with a minor percentage being γ9δ1 T cells. Collectively, these T cells exert potent cytotoxic effects and play key functions in the defense against microbial infections and cancer cells (81, 82). Human γδ T cells are MHC-unrestricted and are immune surveillance cells against tumor and infection. These T cells can recognize stress-related signals. For example, the MHC class-I chain-related proteins (MIC-A/B) and human cytomegalovirus (CMV) membrane glycoprotein-binding proteins (ULBPs) on cancer, and transformed or infected cells (83) can be activated when γδ T cells bind to phosphoantigens (PAgs) such as (E)-4-hydroxy-3-methylbut-2-enyl pyrophosphate (HMBPP), isopentenyl pyrophosphate (IPP) and/or stress-associated antigens *via* the NKG2D receptor. Once activated, γδ T cells secrete abundant cytokines and execute a cytotoxic function (84). Compared to conventional T cells, γδ T cells are resistant to activation-induced T-cell death (AICD), which paves the way for γδ T cells to mount enduring anti-tumor responses. γδ T cells also naturally home to various tissues. Recently it has become feasible for researchers to produce enough cells for cancer immunotherapy due to the recent developments in the methods for robust expansion of γδ T cells. The discovery of activation with phosphoantigens and cytokines such as IL-2 and IL-15 has also improved production (78). These characteristics and the progress of γδ T cells hold manufacturing give hope to a potentially superior approach for eradication of solid tumors in tissues (85).

Early clinical experience with autologous γδ T cell therapy has included pilot studies, Phase I and Phase I/II trials. These investigations first started in 2003 and are ongoing. (We will not discuss in detail the systemic administration of PAgs or N-bis and interleukin (IL)-2 to activate γδ T *in vivo*. This approach is well tolerated.) However, the clinical benefits appear to be modest, likely due to the impairment of γδ T cells and their function, as well as the activation-induced energy and exhaustion of γδ T cells in cancer patients. We will focus our discussion on the first-in-human (FIH) ACT in GBM using *ex vivo*-expanded autologous γδ T cells. The repeated administration of *ex vivo* activated and expanded autologous γδ T cells was completed. (84). The cell therapy product of the FIH γδ T cell therapy trial in GBM is methylguanine DNA methyltransferase (MGMT)-modified γδ T cells expanded *ex vivo* from GBM patients. It has passed the pre-clinical stage (86) and progressed into a Phase I study (87). MGMT can repair damaged DNA to avoid cell death induced by alkylating agents. MGMT plays an important role in increasing chemoresistance to alkylating agents (88). TMZ and chemotherapeutic drugs have been successful in reducing the mass of GBM tumors. They can also transiently upregulate the expression of multiple stress-induced NKG2D ligands (NKG2DL) on GBM cells to sensitize GBM cells to the oncolysis of γδ T cells (89). Meanwhile TMZ suppresses the anti GBM function of the lymphocyte effectors. Modification of γδ T cells with a MGMT transgene gave rise to the TMZ resistance by using lentiviral vector encoding of the DNA repair enzyme O(6)-alkylguanine DNA alkyltransferase (AGT) from the O(6)-methylguanine methyltransferase (MGMT) cDNA (86) This ensures and enables the γδ T cell executing oncolytic function in therapeutic concentrations of TMZ. This gene-modified γδ T therapy approach improved survival outcomes in mouse and xenograft models of primary and refectory GBM circumstances. Briefly, in a PDX model of primary high-grade gliomas models, the concurrent dosing of MGMT-modified γδ T cells and TMZ improve the survival outcomes when compared with single-agent chemotherapy and single agent γδ T cell-based therapies. These preclinical investigations strongly support the rational of developing a “Drug Resistant Immunotherapy” approach for GBM treatment (87). In a phase I clinical trial, the single-dose administration of MGMT genetically modified gamma delta T-cells in lymphodepleted GBM patients was well tolerated. No dose limiting toxicities (DLTs) such as infusion reactions, cytokine release syndrome (CRS), or neurotoxicity were observed. The first treated patient survived 15.6 months post-diagnosis compared to an expected median overall survival term of 10 months given multiple poor prognostic factors. Cohort 2 of the phase 1 study with multiple repeat doses is ongoing (90).

Allogeneic γδ T cells have been identified as one of the critical contributors to the graft vs. tumor (GVT) effect. The GVT effect, one of the most effective anti-cancer immunotherapies, occurs in allogeneic bone marrow/stem cell transplant (BMT) settings. The GVT response can eradicate chemotherapy/radiation resistant tumors. (91). Meanwhile, allogeneic γδ T cells are safely used in haploidentical transplants without risk of GvHD (92). Moreover, allogeneic γδ T cells have strong potency in killing tumor cells because allogeneic γδ T cells are sourced from healthy donors with a full spectrum of natural immune surveillance functions. The use of allogeneic CAR-γδ T cells is a novel strategy to enable the CAR-γδ T cells to eradicate tumors independently on their TCR.

Drug pharmaceutical developers and physicians must avoid severe brain inflammation occurring during ACT for GBM, in particular allogeneic ACT. The severity of the immune response and inflammation during GBM treatment needs to take into consideration several key factors. The items include: the sensitivity of the human brain and the CNS cavity, and the existing increased intracranial pressure with the tumor mass. Thorough consideration must be given to optimizing CAR structure and controlling the character and potency of cell therapy products. This can be done by designing the most feasible and safe delivery route and, treatment regimen: such as the frequency of the treatment, the dosage size of the cell therapy drug, pre-conditioning, possible dose limited toxicity and treatment to reduce local endogenous inflammation; and the minimizing the off-tumor toxicity within CNS and the peripheral tissues.

As in other types of cell therapies, the TME limits the cytotoxicity of γδ T cells by their regulatory function. This is accomplished by the secreting of immunosuppressive cytokines, and by inhibiting immune checkpoint molecules. TME and GBM treatment regimens will impact the plasticity of γδ T cells. γδ T cells secrete TNF-α and IFN-γ upon activation by PAgs mounting a Th1 immune response. γδ T cells are capable of displaying the functionality traits similar to Th2 cells, Th17 cells, or regulatory T cells (Tregs) (93, 94). An effective strategy for overcoming the immunosuppressive effects of the TME is the TME-targeting therapies, such as immune checkpoint inhibitors. The application of these inhibitors can rescue the immunosuppression of TME-constituting cells.

What should also be considered is the fact that cancer stem cells could mediate resistance to γδ T cell immunotherapy. The *ex vivo*-expanded γδ T cells might not be able to eliminate GSCs (95). Designing and developing CAR to target GSCs could be a solution for γδ T cells therapy because by doing so it would be attacking GSCs to reduce a relapse in GBM.

Strategies to increase the safety and enhance the potency of γδ T therapy include 1) Direct delivery of *ex vivo* expanded γδ T cells into a local cavity, such as ICT and/or ICV. This strategy has been shown to reduce systemic toxicity. 2) For clinical applications, combination therapies improve the anti-tumor effects of γδ T cells when applying γδ T cells with anticancer agents, molecularly targeted agents, and epigenetic agents. For example, while treating GBM, TMZ increases the expression of NKG2D ligands on tumor cells, while it increases the γδ T’s oncolysis on the tumor cells (86). 3) The use of a bispecific antibody and/or CAR-transduced γδ T cells will promote current therapeutic efficacy. This strategy enables the CAR-γδ T cells to bind to the tumor epitopes independent of their TCR. There are on-going CAR-γδ T cell trials for hematology and solid tumor (Colorectal Cancer, Triple Negative Breast Cancer, Sarcoma, Nasopharyngeal Carcinoma, Prostate Cancer, Gastric Cancer) applications. We are not aware of a current CAR-γδ T cell trial for the treatment of GBM.

Pharmaceutical developers are actively researching and developing strategies to propagate and control the plasticity of γδ T cells on a large scale in a GMP environment. If successful this will go a long way to accommodating the clinical needs of GBM researchers.

## Natural Killer T (Nkt) Cells

NKT cells are an extremely rare subset of T cells. They typically comprise less than 1% in PB of humans (96). NKT cells simultaneously express a wide spectrum of NK receptors in addition to the αβ T cell receptor. Unlike conventional T cells, NKT cells recognize lipid antigens *via* a CD1d-restricted manner. NKT cells can be grouped into three distinct subsets based on the TCR that they express: type I NKT (classical NKT cells), type II NKT (non-classical NKT cells), and NKT-like cells (CD1d-independent NK1.1+ T cells) (97). Type I NKT cells contribute significantly to anti-tumor immunity. The exogenous agonistic antigens such as α-GalCer activate Type I NKT cells. Type I NKT cells can have direct oncolytic function on CD1d-expressing tumor cells. They can facilitate tumor immunosurveillance and generate endogenous anti-tumor immune responses, including anti-GBM immune responses (98). On the flip side, Type II NKT cells execute an immunosuppressive role in cancers. They also cross-regulate Type I NKT cell activity *via* the IL-13 secretion which prompts myeloid cells to produce TGF-β. Their specific role in GBM remains largely unclear and requires more investigation (99).

NKT cell application in GBM treatment is being researched and is in the early preclinical stage. There are studies demonstrating the killing function of NKT cells on GBM cell lines and the reduction of tumor burden by NKT cells *in vivo* GBM xenograft mice models. While treating intracranial tumors in mice with NKT cells, the additional α-GalCer treatment can increase the survival of the mice. Type I NKT cells mediate killing on CD1d-positive GBM cell lines or CD1d-positive patient-derived GBM cells after the NKT cells are expanded with IL-2 and α-GalCer (KRN7000, a synthetic glycosphingolipid originally isolated from a marine sponge). Researchers noted significant increases in the production of IFN-γ, TNF-α, granzyme B, and IL-4 (100). Type I NKT cells can be functional effectors for the ACT of CD1d expressing tumors. When human type I NKT cells with a-GalCer are intracranially co-injected into tumor-bearing mice with the CDld-positive U251 orthotopic xenogenic in a GBM model, scientists noted a significantly prolonged survival rate. Investigators wanted to observed the delayed tumor growth rate by injecting type I NKT cells with and without α-GalCer to compare to the control injection. In contrast, type I NKT cells failed to hinder tumor growth of CD1d-negative U87 cells in the intracranial injection model. This suggests that human type I NKT cells mount direct cytotoxicity against CD1d-expressing GBM cells. The expression of CD1d in GBM holds the promise of anti-GBM therapeutic potential using NKT cell-based cancer immunotherapy (100).

NKT cells may play a critical role in the brain cancer immune landscape. The human brain contains large amounts of lipid. A brain with GBM generates abnormal lipid metabolisms which in turn generates the accumulation of the aberrant lipids (99, 101). Immune response can be induced by some lipids, such as gangliosides shed from tumor cells. NKT cells recognize the lipids which can be presented by CD1d, such as sphingolipids and glycerophospholipids (102). Even though the investigation of NKT cell therapy treating GBM is in the early pre-clinical stage, we await in great anticipation the developing NKT cell therapy to efficiently treat GBM.

## Monocytes, Dendritic Cells (Dcs) & Macrophages – Myeloid Lineage Immune Cells

In recent years, a number of scientific investigations have focused on myeloid immune cells (Monocytes, DCs and Macrophages) for the treatment of GBM. This is due in part to a distinctive large amount of myeloid immune cells infiltrating the brain’s microenvironment. By producing immunosuppressive and anti-inflammatory cytokines and growth factors, these cells regulate the GBM immune microenvironment and can be associated with tumor progression. They can also promote T-cell apoptosis (103, 104). The critical influence of the TME on the efficacy of GBM immunotherapy suggests that altering myeloid immune cells might be a new strategy for GBM treatment.

Monocytes can migrate into tissues and differentiate between DCs and macrophages (105). Which means that monocytes can successfully cross BBB, and improve GBM outcomes. In an experiment setting, monocytes migrated through an artificial endothelial barrier, penetrated and released drugs in GBM spheroids (106).

In GBM microenvironments, about 30-50 percent of the tumor mass are tumor-associated macrophages (TAMs) and microglia (107). The TAMs’ plasticity toward anti-tumor M1 (inflammatory TAMs) and pro-tumor M2 (anti-inflammatory TAMs) phenotypes is one of the notable attributes. By redirecting immunoinhibitory M2 TAMs into the immunostimulatory M1 phenotype investigators can reduce immunosuppression and boost immunity driven by cytotoxic T lymphocytes (CTLs) (108). Improved patient survival rates were observed when correlated with M1 polarization (109). M2 polarization has been on the opposite correlation (110). Hence, regulating TAMs can be an innovative strategy for GBM therapy (111). CAR Macrophage therapy has demonstrated tumor reduction and prolonged overall survival in xenograft mouse models, and resistance to immune suppressive TME (112). Even though the investigations related to this therapeutic area are still in progress, TAM-targeted immunotherapy has aroused some increased attention in recent years (104).

DCs were described by Ralph Steinman in 1977 (113). DCs are major antigen presenting cells (APCs). DCs process and present antigens to the innate and adaptive immune systems through major histocompatibility complex I and II (MHC I and II) (114, 115). The DC-mediated presentation of GBM-related antigens and peptides for immune cells activation are the successful factors in GBM treatment. The anti-tumor immunity induced in GBM patients and the effectiveness of the DC vaccine in pre-clinical models have been observed by using DCs pulsed with tumor lysates or synthetic peptides (116–118). Substantial promise of prolonged median OS that was seen in the DC treatment group has been shown in early-stage clinical trials (119–121). In 2013, Vik-Mo et al. used a DC vaccine targeting GSCs for treatment. They claimed that seven patients in the study showed a 2.9 times longer progression-free survival (PFS) rate in the vaccine treatment group compared with the matched controls (122). ICT-107 is a double-blind, placebo-controlled Phase II trial for newly diagnosed GBM patients utilizing TAAs that are present on GBM cells. Six synthetic peptides are created and pulsed onto the patient’s DCs. This trial was designed to evaluate the safety and efficacy of TAAs pulsed DCs administered in conjunction with the Stupp protocol (2, 123). The significant increase of PFS (2.2 months) was observed in the ICT-107 cohort in the intent-to-treat (ITT) population (124). In the later Phase II trial, comparing the standard of care, ICT-107 did not show the OS benefit. In 2015, in order to compare the standard of care to ICT-107, a randomized, double-blind Phase III trial was conducted. Unfortunately, due to insufficient financial resources, the study was suspended before reaching its primary outcome. (https://clinicaltrials.gov/ct2/show/NCT02546102).

An autologous DC vaccination phase-II clinical trial evaluated the efficacy of autologous DC pulsed with whole tumor lysate in 27 newly diagnosed GBM patients. The DCs were generated from patients’ PBMC and pulsed with autologous whole tumor lysate. The findings showed that 12.7 months was the median PFS (CI 95% 7-16). Further, 23.4 months (95% CI 16-33.1) was the median OS. The tumor-specific immune response, such as proliferation and/or cytokine release was significantly increased post the vaccines. This finding was shown in 11 of the 27 (41%) evaluated patients. There was no significant correlation between the immune response and the survival rate (125). A Phase III trial was conducted after the Phase II trial in the newly diagnosed GBM patients. The purpose of the Phase III trial was to focus in and evaluate the autologous tumor lysate-pulsed dendritic cell vaccine (DCVax^®^-L) to the standard care. This dendritic cell vaccine was added to the standard therapy for the newly diagnosed glioblastoma. Patients were taken in random order (2:1) after surgery and chemoradiotherapy, to receive temozolomide plus DCVax-L (n = 232) or temozolomide and placebo (n = 99). The intra-dermal injection in the arm was the delivery route for the DCVax-L. The injections were given six times in the first year and twice per year thereafter. Results were reviewed 15-17 months after the start of the surgery. Data were also collected in the trial’s overall intent-to-treat (ITT) patient population. From the time of surgery, mOS in DCVax-L treated patients was 23.1 month comparing positively with the mOS of 15–17 months which was usually achieved by standard of care (SOC) (126). GBM patient survival may be extended with the addition of DCVax-L to standard therapy. The data demonstrated the safety and feasibility of this treatment. The Phase III observation was published in 2018. Sufficient events have not yet been achieved (i.e., patient deaths) to justify or consider unbinding. Taken in whole, the blinded interim survival data suggested that patients who received the DCVax-L treatment could live longer beyond the researchers’ expectations (127).

Intensified temozolomide doses (DI-TMZ) were given to 11 patients with newly diagnosed GBM. The strength of the dosage was 100 mg/m2/d × 21 days per cycle. At a minimum, three vaccines of pp65 lysosome-associated membrane glycoprotein mRNA-pulsed DCs admixed with GM-CSF were administered on day 23 ± 1 of each cycle. The major component of the human cytomegalovirus (CMV) was pp65 (ppUL83). After virus penetration, the dense bodies (noninfectious particles) and the virions localized, for the most part, to the nucleus. (128). If the patients had not progressed with the treatment, a dose of DI-TMZ and pp65-DCs were administered every month. Cellular responses were reported to have increased dramatically after the first cycle of DI-TMZ, and three doses of pp65-DCs. The proportion and proliferation of regulatory T cells (Tregs) increased and continued to be high with routine DI-TMZ cycles. The median PFS of 25.3 month [95% confidence interval (CI), 11.0-∞], and the median OS of 41.1 months (95% CI, 21.6-∞) were reported in this trial. Recursive portioning analysis and matched historical controls were used to determine whether the survival rates exceeded expectations. The number of patients who maintained the status of being free of progression was four after 59 to 64 months from diagnosis. Patients who received pp65-DCs showed long-term PFS and OS, demonstrating cytomegalovirus is a good target in GBM treatments consistent with previous studies. (129).

GBM trials using DC demonstrated the feasibility and safety of this therapeutic approach. Preliminary clinical efficacy is promising, but still only modest in success. Until now, all the trials utilize autologous DC prepared from PBMCs loaded with different antigens, peptides, mRNA or GBM tumor lysate. Autologous DC from GBM patients might already be imprinted with impaired immune signatures which might impact the function and potency of the DC vaccine. The effector cells and signals, such as cytokines and chemokines, induced by DC vaccines still need to find their way to cross BBB, reach the GBM site and overcome the severe immune suppressive TME to play their roles in combating this disease.

## Stem Cells

The use of stem cells and their derivatives has emerged as an innovative strategy to treat GBM. Stem cells, by definition, are the cells with the unique ability to develop into many different cell types. They are also able to self-renew to maintain their stemness. In the human body, there are two major types of stem cells: adult stem cells and embryonic stem cells (ESCs). In recent years, another specific type of artificial stem cell, induced pluripotent stem cells (iPSCs), can be produced by converting somatic cells into a pluripotent stage through the process of reprogramming (130, 131). Over the past 20 years, numerous preclinical studies have demonstrated that using stem cells in GBM therapy can lead to a significant reduction of the tumor size and improve the treatment (132).

### Adult Stem Cells

Adult stem cells can be found in most tissues in postnatal life. They are undifferentiated but lineage-committed cells. Although they are often rare populations in the resident tissues, this special type of cell plays a critical role in replacing cell lost due to tissue turnover or injury, thus maintaining the homeostasis of different tissues. Several types of adult stem cells, including neural stem cells (NSCs), mesenchymal stem cells (MSCs) and hematopoietic stem cells (HSCs) have been tested to treat GBM.

Among adult stem cells, NSCs have shown the greatest potential for GBM therapy. NSCs can be mainly detected in the hippocampus and the subventricular zone of the brain (133). The attraction of using NSCs to treat GBM is that they have the ability to migrate deep into the tumor tissue, thus, they can serve as a vehicle to deliver a variety of therapeutic agents to the tumoral mass (134, 135). The tropic property of NSCs was demonstrated in a study published in 2000 (136). When NSCs were injected directly into the tumor or implanted intracranially at a distance, they could migrate to and be distributed widely throughout the tumor. This migratory ability remained when they were transduced to express a therapeutic transgene encoding the enzyme cytosine deaminase (CD). Simultaneously, another study demonstrated the therapeutic benefit when combining the immune-therapy and NSCs to treat GBM (137). In this study, the gene for the cytokine interleukin-4 (IL-4) was transduced into neural progenitor cells and these cells were injected into GBM tumors. The progressive disappearance of large tumors could be detected several weeks after injection which led to the prolonged survival rate of tumor-bearing mice. In addition to IL4, promising results were also published with the delivery of IL-12 (138) and IL-23 (139) for treatment of GBM. In the latter study (139), the mouse bone marrow-derived neural stem-like cells (BM-NSC) were genetically manipulated to express IL-23. When these cells were injected into intracranial glioma-bearing mice, approximately 60% of these mice survived beyond 120 days and remained tumor-free. When the surviving animals were rechallenged with parental glioma cells, they were resistant to the tumor cells and remained tumor-free which indicated the benefit of long-term antitumor immunity after IL-23-expressing NSC treatment.

Over the years, NSCs have been used as a delivery vehicle to treat GBM. They were not only tested with cytokine gene transduction therapies but also with other strategies. Some of the strategies include the expression of enzymes, proapoptotic molecules, nanoparticles, and oncolytic viral therapies (132). With the development of this field, an NIH study was conducted by Portnow et al. and the results were published in 2017 (140). In this report, 15 patients with recurrent high-grade gliomas underwent intracranial administration of a NSC line with stable expression of cytosine deaminase (CD-NSCs) which could convert the prodrug 5-fluorocytosine (5-FC) to 5-fluorouracil (5-FU). Although this study did not show significant differences in progression-free or overall survival compared to the experiment control cases, it did provide proof of concept that genetically modified NSCs could distribute to targeted areas and that the strategy was relatively safe after transplantation. No dose-limiting toxicity was found; CD-NSCs migrated to distant tumor sites and were nontumorigenic; and they could produce 5-FU locally in brain tumors. To date, there are only six clinical trials recorded in the NIH database (www.clinicaltrials.gov) using NSCs as the tool to deliver carboxylesterase, cytosine deaminase, or oncolytic adenovirus to treat GBM (**Table 2**).

In addition to NSCs, another type of adult stem cell, the mesenchymal stem cell, is being widely tested in the treatment of GBM. MSCs are multipotent stem cells that can be obtained from bone marrow, peripheral blood, adipose tissue, umbilical cord, cord blood, dental pulp, et al. (6). They are spindle-shaped cells which can be relatively easily isolated, expanded extensively during *in vitro* culture while maintaining their potential to generate different types of cells (141). MSCs have the unique advantage of not being immunogenic (142) and have shown potential for autologous transplantation without rejection or GVHD due to lack of MHC-II and only minimal MHC-I expression (143). Evidence from experimental studies demonstrated the strong tumor tropism of MSC. When transplanted into GBM animal models by intra-cerebral, intra-tumoral, intra-venous, or intra-arterial injection, MSCs were shown to be able to migrate to orthotopic GBM tumors (144). This MSC feature was originally demonstrated using fluorescently labeled human bone marrow-derived MSCs when transplanted into a mouse model (145). Recent visible evidence of migration was reported by Kim and colleagues using bioluminescence imaging (146). The easily accessible and expandable source of cells, the tropism to malignant gliomas, and the immune-evasive feature altogether make MSCs a promising cell therapy resource to deliver anti-tumor agents. MSCs have been used by many studies as the cell vector to deliver chemotherapeutics, nuclei acids, immunomodulators, apoptotic agents, oncolytic viruses, or suicide genes. A number of animal studies have demonstrated effective suppression of GBM treated with MSCs, but more data from clinical trials will be needed before MSCs can be used in clinics. (144).

Hematopoietic stem cells are the most well-characterized adult stem cells which can differentiate into different types of blood cells. These stem cells are present in cord blood, adult bone marrow, and mobilized peripheral blood. Several studies showed that HSCs could also be a therapeutic delivery vehicle. In 2005, it was demonstrated that intravenously injected hematopoietic stem/progenitor cells (HSPC) could home to experimental intracerebral gliomas, and this process was mediated by a CXC chemokine ligand (CXCL) 12-dependent pathway (147). When HSCs were genetically manipulated with lentiviral transduction, they didn’t become tumorigenic or change their glioma tropism (148). Another study also demonstrated that when HSPCs were intravenously administered during ACT, they rapidly migrated to sites of malignant glioma growth. They also facilitated the recruitment of tumor-specific lymphocytes into glioma microenvironment thus enhancing ACT efficacy (149). Follow-up studies showed that HSPCs differentiated into potent antigen-presenting dendritic-type cells, led to enhancement of intertumoral T cell activation, and enhanced the immunologic rejection of gliomas (150). In a recent study, HSCs were transduced with a lentiviral vector expressing soluble TGFβ receptor II-Fc fusion protein. When the TGFβ-blocking HSC gene therapy combined with irradiation, it significantly increased the survival rate in tumor-bearing mice compared with the control groups (151). This demonstrated the feasibility of using HSC gene therapy to treat GBM patients in the future.

### Pluripotent Stem Cells

Embryonic stem cells (ESCs) are pluripotent stem cells which can be derived from the inner cell mass of the blastocyst. They have the capacity to self-renew and to differentiate into any somatic cell types. Because of these abilities, ESCs are considered an alternative source to produce downstream differentiated cells to treat various diseases including cancer. For example, ESCs could be engineered with human TRAIL and then directed to differentiate into astrocytes. During *in vitro* coculture, these ESC-derived astrocytes significantly increased the apoptotic rate of human malignant glioma cells (152). When these ESC-derived TRAIL-expressing astrocytes were injected into a mouse model, they induced apoptosis in human malignant gliomas xenografts (153). MSCs could also be differentiated from ESCs (154). Similar to isolated MSCs, these cells could serve as delivery vectors for GBM treatment. They were able to migrate into human glioma xenografts, and with the expression of a transduced thymidine kinase gene, they inhibited tumor growth and prolonged the survival rate of tumor-bearing mice (154).

In 2006, a new type of artificial stem cell, called an induced pluripotent stem cell (iPSC) was generated by delivery of a mixture of reprogramming factors into somatic cells (130). Similar to ESCs, iPSCs have the capacities for indefinite proliferation and multilineage differentiation. Using iPSC-derived cells to treat different human diseases has attracted the research community (155). Only 16 years after its first discovery, more than 30 clinical trials have been registered in the NIH clinical trial database (clinicaltrials.org). In a study published in 2015 (156), Yamazoe and colleagues demonstrated that both iPSCs and iPSC derived-NSCs had similar *in vitro* tropism to glioma-conditioned media. When injected into glioma-cell-implanted mice, both stem cells could migrate to the tumor area which suggested iPSCs and their derivatives can be used as vehicles in glioma therapy (156). When iPSC-derived NSCs were transduced with the suicide gene, herpes simplex virus thymidine kinase (HSV-TK) and then transplanted into a GBM mouse model, tumor growth was dramatically inhibited with significantly prolonged animal survival rates (157). Besides NSCs, iPSCs have demonstrated the ability to differentiate into various immune cells such as T cells (158), NK cells (159, 160), and macrophages (161, 162). These cells have been widely tested to treat GBM as summarized in previous sections. With the great advances in the iPSC field in recent years, it has the potential to allow the use of stem cell-derived products to treat GBM.

## Discussion

Cell Therapy has revolutionized the treatment of multiple diseases, including several kinds of cancer. In this review, we introduced various immune cell- and stem cell-derived adoptive cell therapies in GBM treatment.

Based on each cell type, we can design and develop autologous or allogeneic cell therapy products. We would like to consider the allogeneic therapy option if possible. The individualized autologous cell therapy products pose significant limiting factors for large scale clinical applications. The off-the-shelf, ready-to-use allogeneic cell therapy design can enable scaling up, standardization, automation of the manufacturing process and promote cost reductions. In particular, allogeneic stem cell therapies provide the possibility of controllable, continuous and consistent cell therapy production and significantly reduce the waiting time for GBM patients to receive the advanced treatment. One critical factor for consideration in the selection of a cell therapy is that autologous therapy requires that an initial cell product be generated from GBM patients. The various immune cells in GBM patients might already be imprinted with impaired immune signatures which facilitate tumor growth. On the contrary, we can strictly select healthy donors and cells with beneficial anti GBM potential to produce allogeneic cell therapy products. The feasibility of designing allogeneic cell therapy products will need to be evaluated based on the particular cell type and safety of the regimen.

Innate immune cells have distinct advantages over adaptive immune T cells as candidates for treating GBM because of GBM’s high intratumoral and intertumoral heterogeneity and low mutational burden. Because of GBM’s intracranial location, drug pharmaceutical developers and physicians must carefully consider and balance the efficacy of cell therapy and the immune inflammation induced by the treatment. We should avoid any severe brain inflammation induced with ACT, in particular allogeneic ACT. Pharmaceutical developers and physicians must thoroughly consider and design the most feasible and safe delivery route (IV, ICT, ICV), treatment regimen, such as frequency of the treatment, dose of the cell therapy drug, pre-conditioning, possible dose limited toxicity and treatment to reduce local endogenous inflammation; minimizing the off-tumor toxicity within CNS and the peripheral tissues. Strictly controlling the character and potency of cell therapy products not only can reduce the risk of severe immune inflammation and immune related adverse events, but also it can reduce the variables in the treatment that might contribute to the clinical outcome.

Cell therapy has demonstrated a few successes in GBM treatment so far. The advance cell therapy trials have primarily been investigated on relapse and refractory GBM patients rather than on primary diagnosed GBM patients. We would encourage more evaluation of advanced cell therapies and/or combined therapies in the newly diagnosed GBM. This would produce more efficient therapeutic responses and clinical efficacy. The combination of the advance cell therapies with other approaches, such as small molecule inhibitors, immunotherapy reagents, cell therapies or RNA vaccine might dramatically improve the outcome of GBM treatments.

We also strongly urge researchers to consider investigating biomarkers and mechanisms correlated with the cell therapy process development, clinical treatment and outcome. Through extensive investigations we can stratify the GBM patients into the most efficient treatment regimen at the primary diagnosis stage. Which in turn will optimize and develop the best cell therapy products for the safest, most effective treatment of GBM. The prognosis biomarkers will direct physicians to offer the most promising treatments for GBM patients to consider. We will save lives, improve the quality of GBM patients’ life and also save tremendous resources reducing the health care burden.

The next steps in this journey over the next few years will be both exciting and daunting at the same time. If we are brave enough and inspired enough to accept the challenge, new discoveries will be made that will put an end to the misery of GBM.

The extensive development of cell therapy will realize the full potential of ACT for the treatment of GBM. ACT has the potential to be developed as a routine GBM treatment along with surgery, radiation, and chemotherapy.

## Author Contributions

WW and GW designed and wrote this manuscript.

## Conflict of Interest

Author GW is employed by company BlueRock Therapeutics. Author WW is employed by company Metagenomi.

## Publisher’s Note

All claims expressed in this article are solely those of the authors and do not necessarily represent those of their affiliated organizations, or those of the publisher, the editors and the reviewers. Any product that may be evaluated in this article, or claim that may be made by its manufacturer, is not guaranteed or endorsed by the publisher.
